# Paired electrocatalysis in 5-hydroxymethylfurfural valorization

**DOI:** 10.3389/fchem.2022.1055865

**Published:** 2022-10-21

**Authors:** Dalong Qu, Shuijian He, Lianhua Chen, Yifan Ye, Qingmei Ge, Hang Cong, Nan Jiang, Yang Ha

**Affiliations:** ^1^ Country Enterprise Technology Center of Guizhou Province, Guizhou University, Guiyang, China; ^2^ International Innovation Center for Forest Chemicals and Materials, Co-Innovation Center of Efficient Processing and Utilization of Forest Resources, College of Materials Science and Engineering, Nanjing Forestry University, Nanjing, China; ^3^ National Synchrotron Radiation Laboratory, University of Science and Technology of China, Hefei, China; ^4^ Advanced Light Source, Lawrence Berkeley National Laboratory, Berkeley, CA, United States

**Keywords:** 5-hydroxymethylfurfural, electrocatalytic hydrogenation, electrocatalytic oxidation, paired electrocatalysis, biomass valorization

## Abstract

5-Hydroxymethylfurfural (HMF) has aroused considerable interest over the past years as an important biomass-derived platform molecule, yielding various value-added products. The conventional HMF conversion requires noble metal catalysts and harsh operating conditions. On the other hand, the electrocatalytic conversion of HMF has been considered as an environmentally benign alternative. However, its practical application is limited by low overall energy efficiency and incomplete conversion. Paired electrolysis and highly efficient electrocatalysts are two viable strategies to address these limitations. Herein, an overview of coupled electrocatalytic HMF hydrogenation or hydrogen evolution reaction (HER) with HMF oxidation as well as the associated electrocatalysts are reviewed and discussed. In this mini-review, a brief introduction of electrocatalytic HMF upgrading is given, followed by the recent advances and challenges of paired electrolysis with an emphasis on the integration HMF electrohydrogenation with HMF electrooxidation. Finally, a perspective for a future sustainable biomass upgrading community based on electrocatalysis is proposed.

## Introduction

The rapid development of human civilization and growth of world population result in fiercely global energy demands (Corma et al., 2007; Mika et al., 2018). Due to the declining fossil fuel reserves and the increasing concerns about environmental impacts resulting from fossil fuel combustion, more efforts have been devoted to exploring sustainable energy sources and renewable carbons for organic chemical production (Bozell, 2010; Shi and Zhang, 2016). As the most abundant natural carbon, biomass possesses a great promise in developing carbon-neutral economy (Demirbas, 2001; Hu et al., 2012; Morais et al., 2015). Recently, a biomass-derived chemical, 5-hydroxymethylfurfural (HMF), which is among the Department of Energy’s “Top 10 + 4” list, has been considered as a versatile platform molecule (Bozell and Petersen, 2010; Adeogun et al., 2019; Baga, 2020). Owing to the hydroxymethyl and formyl functional groups attached to the furan ring, further upgrading HMF can generate various high-valued chemicals *via* oxidation, reduction, hydrogenation, esterification, hydrolysis, and cleavage (Binder and Raines, 2009; Bozell and Petersen, 2010; Du et al., 2011; Rosatella et al., 2011; Alamillo et al., 2012; Balakrishnan et al., 2012; Gallo et al., 2013).

**FIGURE 1:**
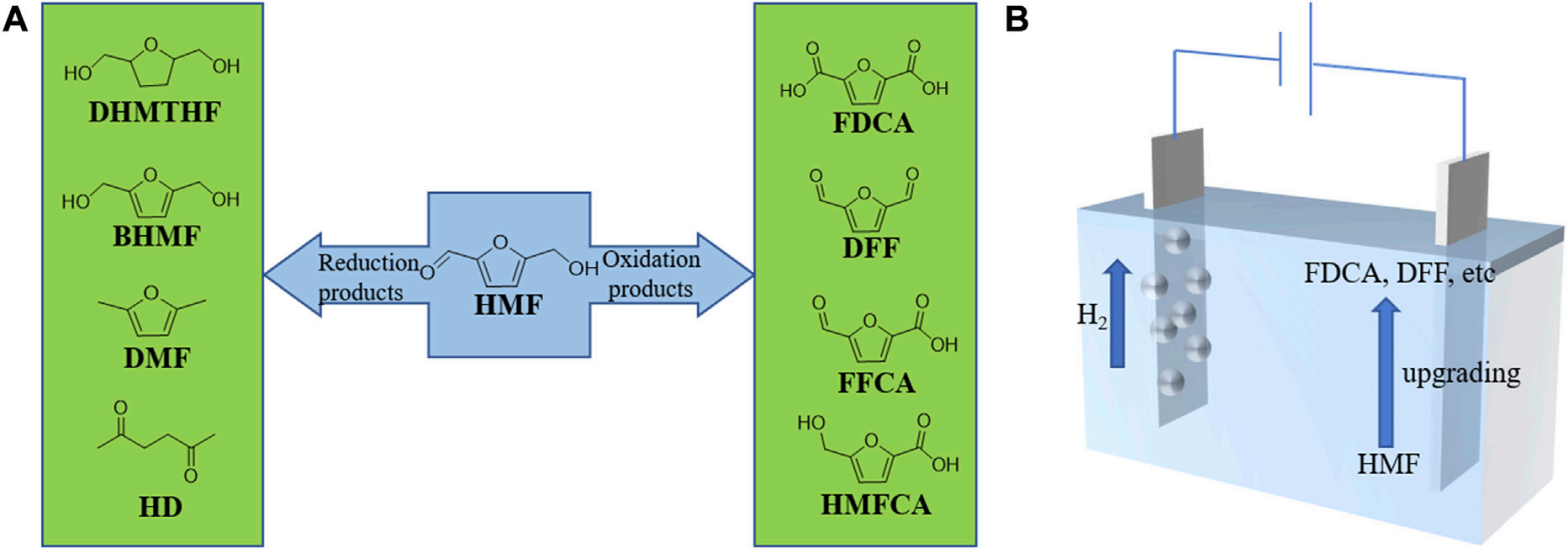
**(A)** Reductive and oxidative products of HMF upgrading. **(B)** Paired electrolysis of HMF oxidation with HER.

The oxidation of HMF yields valuable chemicals, such as 2,5-diformylfuran (DFF), 5-formyl-furan carboxylic acid (FFCA), 5-hydroxymethyl-2-furan carboxylic acid (HMFCA), and 2,5-furan dicarboxylic acid (FDCA), as shown in Figure 1A (Xiang et al., 2011; Vuyyuru and Strasser, 2012a; Antonyraj et al., 2013). Due to the symmetric structure of functional groups, both DFF and FDCA as monomers have tremendous potentials for synthesizing biomass-derived drugs and antifungal agents, furan-urea resins, and other important polymer materials (Liu and Zhang, 2016; Lei et al., 2020). FDCA can serve as a replacement for the petroleum-derived terephthalic acid, producing polyethylene terephthalate and poly (ethylene 2,5-furandicarbocylate) (Wang J. et al., 2017a). In addition, FDCA has a large potential to take a place of terephthalate and butyleneterephthalate, which are used widely in producing various polyesters (Gandini et al., 2009; Kong et al., 2018). Meanwhile, the reduction products of HMF include 2,5-dimethylfuran (DMF), 2,5-dihydroxymethylfuran (BHMF), 2,5-bishydroxymethyl-tetrahydrofuran (DHMTHF), and 2,5-hexanedione (HD) (Connolly et al., 2010; Kong et al., 2014; Scholz et al., 2014; Roylance and Choi, 2016b; Elangovan et al., 2016; Guo et al., 2016; Perret et al., 2016; Luo et al., 2017). Among these reductive products, BHMF, which has two hydroxymethyl groups fused with the furan ring, can also act as a precursor to form polyesters and polyurethane foams (Lecomte et al., 1998; Gandini, 2011; Delidovich et al., 2016; Hu et al., 2018). Moreover, DMF and DHMTHF have been regarded as promising next-generation biofuels (Goyal et al., 2016; Xia et al., 2018). Specifically, DMF has higher energy density and better miscibility than fuel ethanol, which can be used as a potential high-quality liquid biofuel to replace gasoline derived from fossil fuels (Goyal et al., 2016; Kwon et al., 2016). Furthermore, HD is able to serve as the raw material for the production of paraxylene, which is an important precursor to produce polyethylene terephthalate (PET) as well (Roylance and Choi, 2016b). Apart from the above, HMF itself can also undergo hydroxyl-aldehyde condensation with acetone to produce liquid fuel intermediates (Su et al., 2020).

The conventional thermocatalytic valorization of HMF always requires noble metal catalysts (Au, Pd, Pt, and Ru) and harsh operational conditions, such as high temperatures and pressures (Taarning et al., 2008; Casanova et al., 2009; Gorbanev et al., 2009; Davis et al., 2011; Villa et al., 2013; Zhang and Deng, 2015). Additionally, HMF oxidation *via* conventional approaches relies on using toxic oxidants or organic solvents (CH_2_Cl_2_, C_6_H_5_CH_3_, CH_3_CN, etc.) (Amarasekara et al., 2008; Cao et al., 2017). For instance, Gao et al. utilized toxic organic reagents such as methylene chloride and acetonitrile in the process of oxidizing HMF into FDCA (Gao et al., 2015). On the other hand, the conventional methods for the reduction of HMF usually perform at high H_2_ pressure and requires catalysts containing precious metals as well (Alamillo et al., 2012; Wang T. et al., 2017b). There are certain safety hazards when using H_2_ as the proton source for HMF reduction (Bloor et al., 2016). Plus, it is not cost-efficient because H_2_ is a valuable energy carrier. Therefore, it is necessary to explore economical and environmentally friendly strategies to upgrade HMF (Ohyama et al., 2012; Ohyama et al., 2013; Kong et al., 2014; Yang et al., 2017; Sajid et al., 2018). To date, the electrocatalytic HMF upgrading has attracted a great deal of attention (You et al., 2016b; Zhang L. et al., 2019b; Zhang Y.-R. et al., 2019e; Taitt et al., 2019; Kisszekelyi et al., 2020; Zhou et al., 2021). Electrocatalysis is driven by electricity and can be conducted under aqueous and ambient conditions, which is more environmentally benign. Notably, H_2_O can be used as oxidant or proton source instead of costly oxidizing agents and H_2_ gas. More to the point, the reaction rate and product selectivity can be easily controlled by the applied voltage and current (Nilges and Schroeder, 2013; Cha and Choi, 2015; Roylance et al., 2016). In order to achieve higher Faradic efficiency (FE) and overall conversion efficiency, the valorization of HMF can also be paired with other half reactions. From the last 5 years, the research on the paired electrolysis of HMF is mainly focused on coupling the oxidation of HMF with hydrogen evolution reaction (HER), while the combination of HMF reduction with other reactions has received only a few studies. In this mini-review, we summarized the recent advances of paired electrolysis in HMF valorization, which is different from previous reviews. Firstly, we introduce the electrocatalytic oxidation of HMF integrated with HER. Then, the electrochemical reduction strategy of HMF is briefly introduced, followed by emphasizing the upgrading of HMF on both cathode and anode simultaneously. Finally, we discuss the challenges and future directions of paired electrolysis in HMF valorization.

## Paired HMF oxidation with HER

H_2_ is a pollution-free fuel with high energy density, which has been considered as an alternative to fossil fuels (El-Emam and Ozcan, 2019; Xia et al., 2020). Electrocatalytic water splitting to produce clean H_2_ has gained increasing attention (Yu et al., 2018). However, the anodic reaction, oxygen evolution reaction (OER), is the bottleneck of water splitting, which results in the low energy conversion efficiency because of its sluggish kinetics (Zhang J.-Y. et al., 2019a; You et al., 2019). Besides, the product of OER, O_2_, does not have a significantly commercial value. Therefore, replacing OER with a thermodynamically more favorable HMF oxidation reaction can not only solve the safety hazard caused by hydrogen and oxygen mixing, but also improve the overall energy conversion efficiency (Chen et al., 2014; Jiao et al., 2015; Yang and Mu, 2021). As an innovative strategy (Figure 1B), the electrocatalytic oxidation of HMF coupled with HER can produce highly valuable products on both anode and cathode simultaneously (You and Sun, 2018; Dubale et al., 2020). Consequently, it is highly attractive to develop efficient electrocatalysts, especially bifunctional catalysts, to integrate HMF oxidation with HER in a single electrolyzer.

Nobel metal catalysts and their alloys have been extensively studied for electrooxidation of HMF due to their high activity in many chemical processes (Kokoh and Belgsir, 2002; Vuyyuru and Strasser, 2012a; Xu et al., 2019; Du et al., 2020; Park et al., 2020). In addition, noble metals such as Pt and Pd have excellent HER performance. Therefore, noble metal catalysts have been applied as bifunctional catalysts in paired electrocatalysis of HMF oxidation and HER. Kim’s group found that the (AuPd)_7_ alloy had remarkable catalytic performance for electrocatalytic oxidation of HMF and HER in a coupled cell (Park et al., 2020). Although it is hard to realize industrial applications with noble metal catalysts because of their high price, they have contributed a lot to the study of the mechanism of HMF oxidation (Cha and Choi, 2015; Latsuzbaia et al., 2018; Heidary and Kornienko, 2019).

Although noble metals have exhibited excellent catalytic activity for HMF oxidation, they still suffer from high-cost due to their scarcity. Thus, the development of earth-abundant electrocatalysts with high efficiency has become a focus for large scale HMF oxidation integrated with HER. To date, various transition metals ranging from Ni, Co, Cu, Fe to Mn are used to design bifunctional catalysts for HMF oxidation and H_2_ production (Jiang et al., 2016; You et al., 2017; Liu et al., 2018; Nam et al., 2018; Li S. et al., 2019b). Ni (You et al., 2017) and its nitrides (Zhang N. et al., 2019c), borides (Barwe et al., 2018; Zhang P. et al., 2019d), phosphides (You et al., 2016a; Li M. et al., 2019a), oxides (Choi et al., 2020; Gao et al., 2020; Lu et al., 2020), and hydroxides (Latsuzbaia et al., 2018; Chen et al., 2019) have been reported for the electrochemical oxidation of HMF. Among these catalysts containing nickel, NiN_3_@C, Ni_2_P, hp-Ni (3D hierarchically porous nickel-based catalyst), and NiSe@NiO_x_ core-shell nanowires had been used as bifunctional catalysts for both HMF oxidation and HER with high FE (>95%) for FDCA and H_2_, respectively. Moreover, all of these bifunctional catalysts form high-valent nickel species during electrolysis of HMF oxidation. On the other hand, the electrooxidation of HMF can be paired with HER *via* different catalysts as well. For instance, Deng et al. synthesized a “Nanoplatelet-on-Nanoarray” nickel-cobalt hydroxide-based catalyst (t-NiCo-MOF) by simple conversion of a bimetallic metal-organic framework (MOF) nanoarray (Deng et al., 2020b). They used t-NiCo-MOF as the anodic catalyst and MoNi_4_ as the cathodic catalyst to co-generate FDCA and H_2_ at a low voltage of 1.392V vs. RHE with a high current density of 100 mA/cm^2^, which was ∼300 mV lower than overall water splitting.

As competent bifunctional electrocatalysts for overall water splitting, Co-based catalysts have also been employed for coupled electrolysis in HMF oxidation and HER (Jiang et al., 2016; Kang et al., 2020). As early as 2016, Sun and co-workers (Jiang et al., 2016) reported an electrodeposited Co-P as the bifunctional electrocatalyst for integrated HMF oxidation and H_2_ evolution in a membrane-divided electrolyzer, which achieved nearly unity FE and selectivity for both H_2_ and FDCA production. Co_3_O_4_ nanowires have also been studied as bifunctional catalysts for HMF oxidation coupled with HER (Zhou et al., 2019). Surprisingly, the high concentration of HMF (100 mM) was realized by Co_3_O_4_ nanowires, which far surpassed the HMF concentration previously reported. In 2018, Weidner et al. investigated a series of cobalt-metalloid alloys (CoX; X = P, Si, B, As, Te) as electrocatalysts for the oxidation of HMF (Weidner et al., 2018). Among them, CoB showed the highest catalytic performance, which not only had high selectivity for FDCA but also suppressed the decomposition of HMF in alkaline electrolyte.

In addition to Ni- and Co-based catalysts, other transition metals, such as Mn-, Fe-, and Cu-based catalysts have also been reported as competitive catalysts for HMF oxidation. Choi and Kubota used MnO_x_ as an anodic catalyst to achieve the conversion of HMF to FDCA in H_2_SO_4_ solution (pH = 1), showing the possibility of FDCA production in acidic media, which is beneficial to integrate with HER and solve the problem of incompatible electrolyte (Kubota and Choi, 2018). Only a few Fe containing catalysts display high catalytic activity for HMF oxidation. NiFe layered double hydroxide (LDH) was utilized for HMF oxidation, achieving 99.4% FE for HMF conversion (Liu et al., 2018). Meanwhile, they used the benchmark HER catalyst, Pt, as the cathode to produce H_2_. Cu has excellent conductivity and is relatively inactive to water oxidation, which may achieve HMF oxidation with higher efficiency when acted as electrocatalysts (Nam et al., 2018). A typical example is that Cu_x_S@NiCo-LDH core-shell nanoarrays, which approached a current density of 10 mA/cm^2^ at a voltage of 1.34V vs. RHE, yielding nearly unity FE towards both FDCA and H_2_ (Deng et al., 2020a).

## Paired HMF reduction with HMF oxidation

The reduction of HMF is mainly producing biofuels (DMF and DHMTHF), polymer precursors (BHMF and HD), and various organic solvents (Roylance and Choi, 2016b; Goyal et al., 2016; Hu et al., 2018; Xia et al., 2018). The diversity of the products of HMF reduction have obtained extensive research interest due to their wide application prospects (Roylance and Choi, 2016a; Zhang L. et al., 2019b; Zhang Y.-R. et al., 2019e). However, compared with the electrocatalytic oxidation of HMF, the electrochemical reduction of HMF is still at its early stage. In 2013, the electrocatalytic reduction of HMF was first studied by Koper’s group using a series of pure metal electrodes under neutral conditions (Kwon et al., 2013). Subsequently, they investigated the catalytic effects of these metals in acidic solutions and found that the overpotentials for HMF hydrogenation in acidic electrolyte were much lower than that in neutral solutions (Kwon et al., 2015). Among those pure metal electrodes, Ag electrode showed the highest selectivity and conversion for the formation of BHMF. Later, Chio and co-workers modified the silver electrode through galvanic displacement method (Ag_gd_) for electrocatalytic HMF reduction (Roylance et al., 2016). The resulting Ag_gd_ approached high yield (99%) and FE (99%) for BHMF at -1.3 V vs. Ag/AgCl in a slightly alkaline solution. However, electrochemical reduction of HMF is typically paired with OER, which has sluggish kinetics and the unvalued product (Han et al., 2020). Therefore, coupling HMF reduction with HMF oxidation is able to obtain two value-added products and avoid the slow kinetics of OER.

**FIGURE 2:**
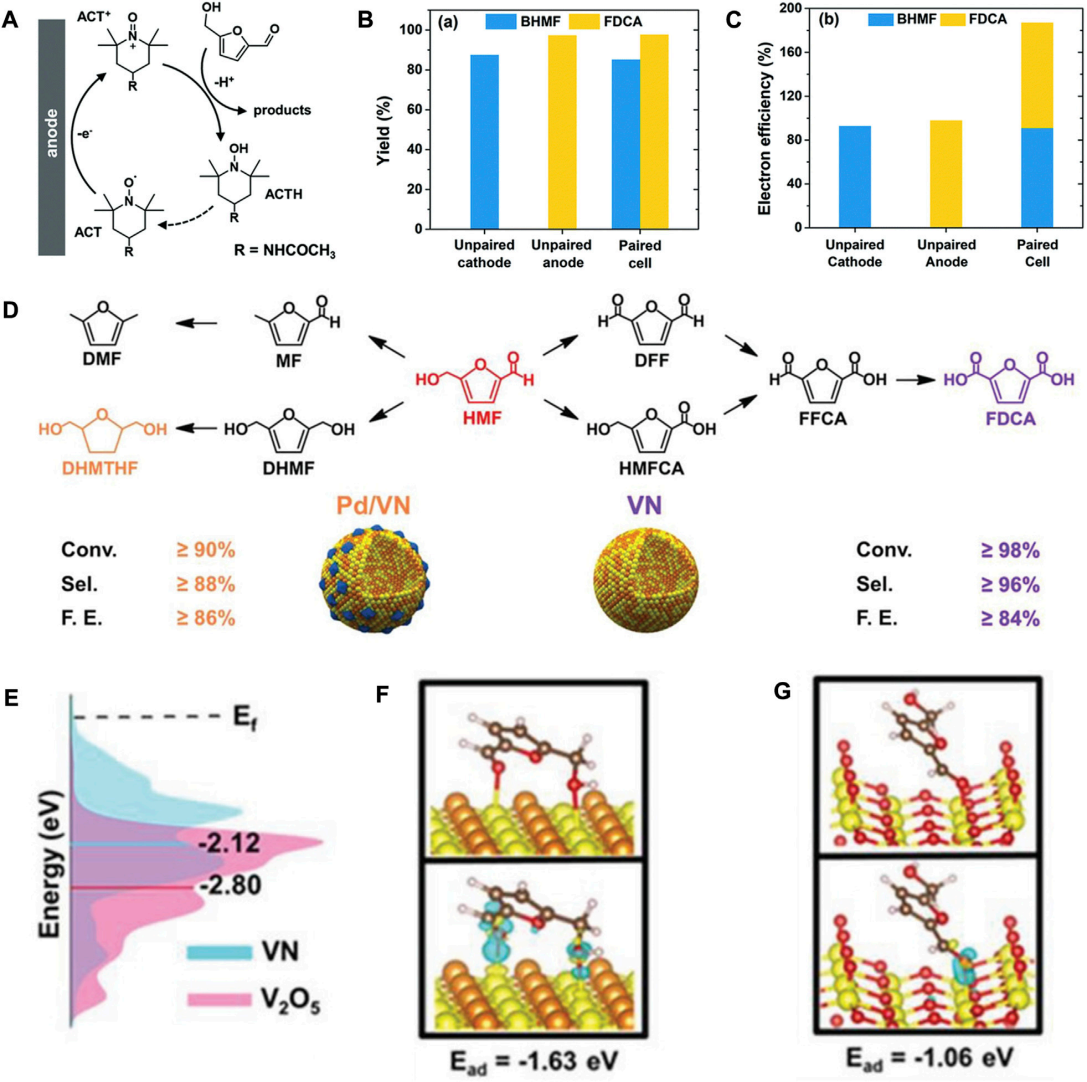
**(A)** Schematic diagram of the ACT-mediated electrocatalytic oxidation of HMF. **(B)** The yields of BHMF and FDCA in unpaired and paired cells. **(C)** The electron efficiencies of BHMF and FDCA in unpaired and paired cells. Adapted with permission from Chadderdon et al. (2019). Copyright 2019 Royal Society of Chemistry. **(D)** Electrocatalytic hydrogenation (left) and electrocatalytic oxidation (right) of HMF over Pd/VN and VN electrocatalysts, respectively. **(E)** Density of states plots of VN and V_2_O_5_. **(F,G)** Optimized structure (up) and charge density difference (bottom) of HMF on VN and V_2_O_5_, respectively. Adapted with permission from Li et al. (2019). Copyright 2019 John Wiley & Sons, Inc.

Although replacing OER with HMF oxidation is a feasible strategy, there are still several challenges which need to overcome to develop paired electrolysis. For instance, the optimal potentials and current densities of the two half-reactions in paired electrocatalysis are unmatched. The well-developed redox mediators are suitable solutions for mismatched problems in paired electrolysis. It was reported that TEMPO (2,2,6,6-tetramethylpiperidine-1-oxyl) and its derivatives (like ACT, 4-acetamido-TEMPO) can work as redox mediators for the electrocatalytic HMF oxidation in mildly alkaline electrolytes due to their rapid redox kinetics, high solubility in water, remarkable stability, and suitable redox potentials (Cha and Choi, 2015; Cardiel et al., 2019). As aforementioned, Ag-based electrode demonstrated excellent catalytic capacity for electrohydrogenation of HMF to BHMF in the same electrolyte (Roylance et al., 2016). Therefore, Li’s group utilized Ag nanoparticles immobilized on carbon black (Ag/C) as the catalyst for electrocatalytic reduction of HMF to BHMF under cathodic conditions. On the other hand, ACT acted as the redox mediator for HMF oxidation (the mechanism is shown in Figure 2A) on carbon felt electrode (Chadderdon et al., 2019). Thus, the ACT-mediated indirect electrooxidation of HMF is insensitive to the anode potentials. With the precise control of the cathode potentials, it was feasible to couple the electrohydrogenation of HMF to BHMF with the oxidation of HMF to FDCA in a single divided cell. The paired electrolysis of HMF achieved 85% yield for BHMF and 98% yield for FDCA, respectively, as well as a combined electron efficiency of 187%, which is the highest electron efficiency for HMF conversion (Figures 2B,C).

In the same year, Wang and co-workers successfully fabricated 3D vanadium nitride (VN) and Pd/VN hollow nanospheres (Figure 2D) as the anode and cathode, respectively, to electrocatalytically upgrade HMF into FDCA and DHMTHF in a bipolar membrane-divided electrolyzer (Li S. et al., 2019b). After electrolysis at 100 mA for 3 h, the conversion of HMF oxidation and reduction was 92% and 87%, respectively, with a high combined FE of ≥170%. For the unpaired HMF oxidation catalyzed by 3D VN, high conversion of HMF (≥98%) was obtained with the high selectivity (≥96%) and FE (≥84%) for FDCA after eight cycles. Compared to other vanadium-based catalysts, such as V_2_O_5_ and VOOH, the high performance of VN can be assigned to its low d-band center (Figure 2E), which facilitate the chemisorption and activation of HMF on VN surface (Figures 2F,G). For the unpaired electrocatalytic hydrogenation of HMF, the high selectivity (≥88%) and FE (≥86%) for DHMTHF were achieved with the help of 3D hollow Pd/VN. Notably, the Pd/VN catalyzed hydrogenation product is DHMTHF, which is different from the previously reported results. Additionally, the 3D hollow structure of electrocatalyst favors the diffusion and transport of substrates.

## Summary and perspective

Recently, paired electrolysis has been widely investigated. In this mini-review, we have overviewed and focused on the recent progress of electrocatalytic oxidation of HMF paired with HER or HMF reduction. In paired cells, value-added products can be obtained on both anode and cathode simultaneously *via* electrocatalytic oxidation and hydrogenation, achieving a combined efficiency greater than 100%. Ideally, utilization of bifunctional electrocatalysts in paired electrolysis is more attractive due to its low cost and facile cell design. Mediated paired electrolysis is another strategy to solve the potential mismatch issues. In general, HMF can be oxidatively converted into DFF and FDCA over monometallic and bimetallic electrocatalysts, including noble metals and transition metals. The intrinsic nature of electrode has a great effect on the pathway of HMF oxidation. With the respect to the reductive upgrading of HMF, Ag exhibits remarkable selectivity to BHMF in slightly alkaline solutions. Overall, optimizing the performance of electrocatalysts to enhance their selectivity, catalytic activity, and stability, is still the main challenge. Although different electrodes have a strong influence on product selectivity and reaction pathway, other reaction conditions, such as mismatched potential and incompatibilities of electrolytes for the two half-reactions, product separation, and crossover issues will also limit large-scale applications for paired electrolysis. More efforts have been devoted to solve the aforementioned problems for industrial application. Additionally, theoretical simulations and *in-situ*/*ex-situ* characterization need to be performed to reveal the reaction mechanisms which are beneficial to design advanced catalysts.

Besides HER and HMF reduction reaction, the oxidation of HMF can also be coupled with CO_2_ reduction reactions (CO_2_RR), N_2_ reduction reactions (N_2_RR), and other organic reduction reactions (Zhang P. et al., 2019d; Xu et al., 2019; Choi et al., 2020). However, these pair-wise electrolysis studies are limited, and more electro-reductive coupling reactions (such as NO_3_^−^, NO reduction, etc.) for HMF should be considered. In addition, photoelectrolysis and bioelectrocatalysis have been also considered as promising alternatives for biomass upgrading (Roylance et al., 2016; Ozcan et al., 2017; Ma et al., 2018; Chen et al., 2020; Meng and Li, 2021; Meng et al., 2022). Moreover, the combination of electrocatalysis and biocatalysis for biomass upgrading can provide yields and selectivity that chemical catalysis cannot achieve (Schmitz et al., 2019).
