# Supplementary figures and images for: Paired electrocatalysis in 5-hydroxymethylfurfural valorization
Source: Front Chem. 2022 Oct 21;10:1055865. doi: 10.3389/fchem.2022.1055865 (PMC9634479; doi:10.3389/fchem.2022.1055865)

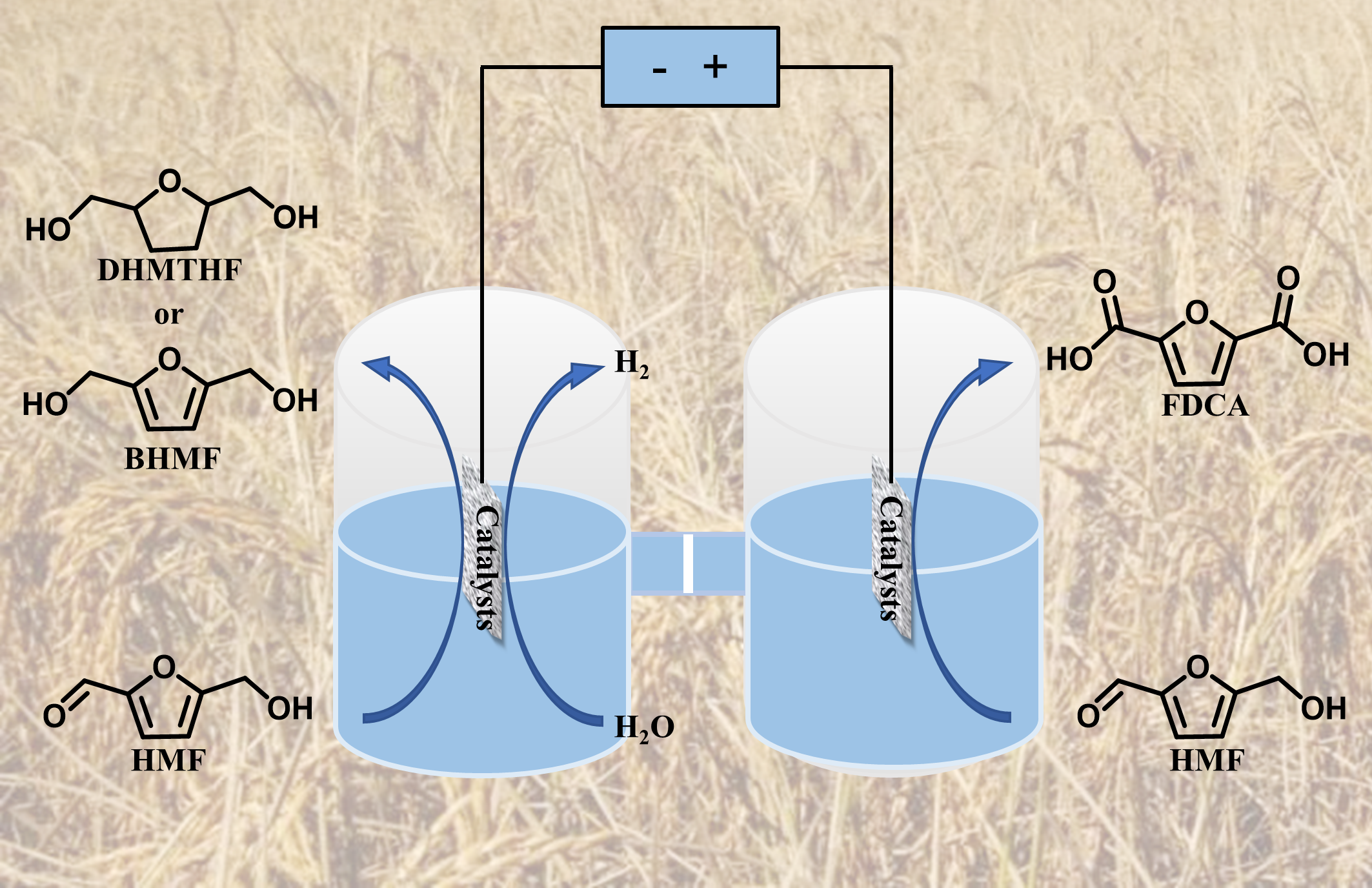

Supplement: Supplementary file 1 [file Image2.TIF]

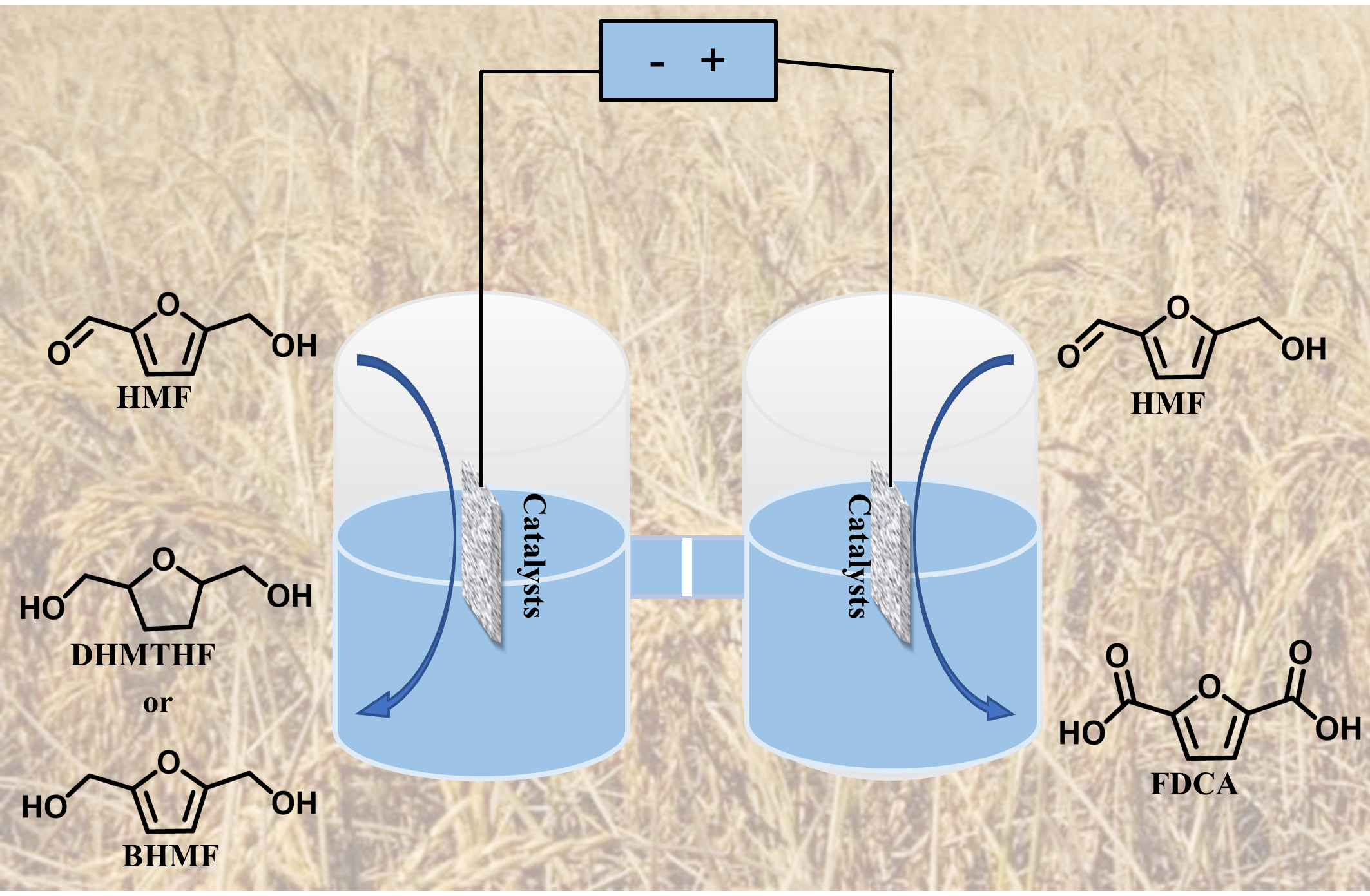

Supplement: Supplementary file 2 [file Image1.TIF]
